# MDU-Net: A Convolutional Network for Clavicle and Rib Segmentation from a Chest Radiograph

**DOI:** 10.1155/2020/2785464

**Published:** 2020-07-17

**Authors:** Wenjing Wang, Hongwei Feng, Qirong Bu, Lei Cui, Yilin Xie, Aoqi Zhang, Jun Feng, Zhaohui Zhu, Zhongyuanlong Chen

**Affiliations:** ^1^Department of Information Science and Technology, Northwest University, Xi'an 710127, China; ^2^State-Province Joint Engineering and Research Center of Advanced Networking and Intelligent Information Services, School of Information Science and Technology, Northwest University, Xi'an 710127, Shaanxi, China; ^3^Chest Hospital of Xinjiang Uyghur Autonomous Region of the PRC, Xinjiang Uygur Autonomous Region, Urumqi 830049, China

## Abstract

Automatic bone segmentation from a chest radiograph is an important and challenging task in medical image analysis. However, a chest radiograph contains numerous artifacts and tissue shadows, such as trachea, blood vessels, and lung veins, which limit the accuracy of traditional segmentation methods, such as thresholding and contour-related techniques. Deep learning has recently achieved excellent segmentation of some organs, such as the pancreas and the hippocampus. However, the insufficiency of annotated datasets impedes clavicle and rib segmentation from chest X-rays. We have constructed a dataset of chest X-rays with a raw chest radiograph and four annotated images showing the clavicles, anterior ribs, posterior ribs, and all bones (the complete set of ribs and clavicle). On the basis of a sufficient dataset, a multitask dense connection U-Net (MDU-Net) is proposed to address the challenge of bone segmentation from a chest radiograph. We first combine the U-Net multiscale feature fusion method, DenseNet dense connection, and multitasking mechanism to construct the proposed network referred to as MDU-Net. We then present a mask encoding mechanism that can force the network to learn the background features. Transfer learning is ultimately introduced to help the network extract sufficient features. We evaluate the proposed network by fourfold cross validation on 88 chest radiography images. The proposed method achieves the average DSC (Dice similarity coefficient) values of 93.78%, 80.95%, 89.06%, and 88.38% in clavicle segmentation, anterior rib segmentation, posterior rib segmentation, and segmentation of all bones, respectively.

## 1. Introduction

Organ and tissue segmentation is essential in medical image preprocessing systems. Automatic rib and clavicle segmentation from chest radiographs are a challenging work. Some lesions are overlooked because they are occluded by bone shadows [1–3]. This finding suggests that by segmenting the ribs in a chest X-ray and then suppressing them, the diagnostic accuracy of lung diseases can be considerably improved [2, 4]. Methods to segment ribs in chest X-ray images have recently been reported. In 2016, Cong Lin et al. proposed the combination of the generalized Hough transform with the bilateral dynamic programming algorithm for rib segmentation [5]. Wu and Guodong proposed rib segmentation by Gaussian filtering, multiscale wavelet analysis, and SVM (support vector machine) algorithms [6]. These methods use traditional handcrafted features to perform segmentation tasks. Most techniques are based on reasonable assumptions for convenience. For instance, the ribs are assumed to be almost equal in width; however, this assumption is almost nonexistent in reality. In addition, these methods only segment relatively clear posterior ribs that overlap with the lungs. The front and rear ribs that do not overlap with the lungs are not segmented. However, these unclear bone shadows largely influence the accuracy of diagnosing pulmonary disease because they are more similar to lesions than easily segmented bones. Simultaneously, to facilitate the localization of lesions (automatic numbering of ribs following the segmentation and using rib numbers to describe the location of the lesion) in lung disease detection systems and the measurement of medical values, such as rib spacing, in automated report generation systems, the complete set of clavicles and ribs has to be segmented from chest radiographs. We distinguish between the anterior and posterior parts of the ribs in actual settings. This separation is not anatomically relevant, and the reason behind the separation is rather technical and specific to X-rays.

Contrast in chest radiography tends to be low and contains numerous artifacts and shadows from other tissues, limiting the high accuracy of traditional methods. Deep learning has recently performed well in natural image segmentation [7], medical image segmentation [8], and video segmentation [9–11]. In medical imaging, deep learning not only excels in tissue and organ segmentation, such as pancreas segmentation [12–14], lung segmentation [15–19], and brain segmentation [20, 21] but also exhibits superior performance in the segmentation of lesions, such as brain tumor segmentation [22–24], brain glioma segmentation [25], and microcalcification segmentation [26]. However, no studies regarding deep learning for rib segmentation have been reported. The main reason is that labeling of ribs requires considerable expertise, resulting in an inadequate dataset. The insufficiency of the annotated dataset hinders the application of deep learning for clavicle and rib segmentation. To address this gap, we have constructed a dataset of chest X-rays and proposed a network framework and then compared our method with the existing semantic segmentation network. The experimental results prove that the proposed networks exceed the performance of other segmentation networks. The main contributions of this study are as follows:

1. A dataset of chest radiograph for segmenting the clavicles and ribs: the dataset consists of 88 cases, each of which contains corresponding mask images of clavicles, anterior ribs, and posterior ribs with a resolution of 1024 pixels × 1024 pixels (weight × height).
2. A multitask dense connection U-Net (MDU-Net) to segment the complete set of ribs and clavicles in a chest radiograph: in the structure, the reuse mechanism is used for feature extraction because of the limited dataset to improve the performance of the network.
3. A feature separation network to solve the multilabel semantic segmentation problem: similar to the classification task, a multilabel problem occurs when a pixel has more than one label.
4. A mask coding mechanism: this mechanism allows the network to learn the characteristics of the foreground and background simultaneously and ultimately improves the segmentation accuracy while avoiding the threshold selection. The average DSC values of 93.78%, 80.95%, 89.06%, and 88.38% are thus achieved in four tasks, namely, clavicle segmentation, anterior rib segmentation, posterior rib segmentation, and segmentation of all bones, respectively.

The remainder of this paper is organized as follows.  Section 2 provides and elaborates description of the proposed approach.  Section 3 presents the detailed experimental setup, experimental results, and analysis.  Section 4 summarizes our study.

## 2. Method

**Figure 1:**
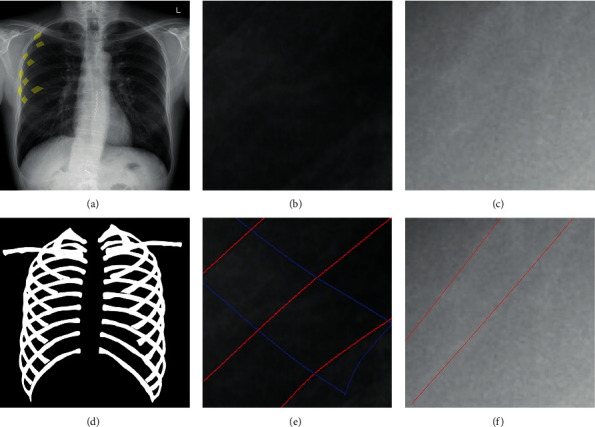
Raw chest X-ray and corresponding marked images. The first column shows the raw chest radiograph and the corresponding mask image. The second column presents a partial enlarged view of the lung area and the corresponding rib border markers. The third column provides a local magnification of the non-lung area and corresponding rib border markers. The edges of the anterior and posterior ribs are marked with blue and red lines, respectively. (a) Raw chest X-ray. (b) Partial enlarged view of the lung area. (c) Local magnification of the non-lung area. (d) Mask image. (e) Rib border markers. (f) Rib border markers.

This study mainly aims to segment the complete set of ribs and the clavicles from the chest X-ray to assist the computer and the physician in diagnosing a disease. As shown in Figure 1, a typical chest radiograph contains not only the clavicle, ribs, lungs, trachea, and spine but also a large number of lung veins and other parts of the body. These anatomical parts hamper the segmentation results. Second, the part marked in yellow in Figure 1 is the overlap of the anterior ribs and the posterior ribs. This phenomenon means that the labeled pixels have multiple tags, which is a difficult problem of traditional segmentation and classification. Such areas are common in chest X-rays. Moreover, we intercept an area overlapped with the lung and another area not overlapped with the lung from the raw chest X-ray and mark the edges of the anterior ribs and the posterior ribs with blue and red lines, respectively. The partial zoomed image shows that the ribs, particularly the anterior ribs, do not have a clear edge and the gray values of the internal pixels are uneven. Outside the lungs, the contrast between the ribs and other tissues is extremely low. These findings indicate that segmenting the complete ribs from the X-ray chest is a highly challenging task and increases the difficulty of rib segmentation and prevents traditional segmentation from achieving good results. Therefore, we propose the design of a network to segment the complete set of clavicles and ribs from the chest X-ray.

### 2.1. Network Structure of MDU-Net

The segmentation task requires both the deep and shallow features, such as edge details, of the segmented objects in the image. With this considered, our network adopts the structure of encoding-decoding to obtain features of different scales. In addition, the huge success of the U-Net network prompts us to improve the results of the decoder by using the feature map of the encoder. In this process, we added feature adaptation (FA) to change the number of channels of features from encoders for feature fusion. This approach reduces the number of channels and balances the weights between feature maps.

**Figure 2:**
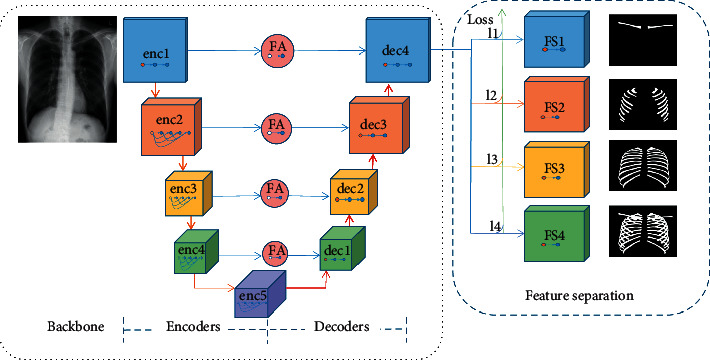
Overview of our architecture for MDU-Net. The network includes backbone network and feature separation network. The backbone network uses an encoder-decoder structure. A feature adaptation layer is added between the encoder and the corresponding decoder to change the number of channels of the feature map to facilitate feature fusion. The feature separation network has four branches, which extract the features required by different tasks and obtain the corresponding segmentation results.

As shown in Figure 2, our semantic segmentation network consists of a backbone network and a feature separation network. The backbone network has 5 encoders and 4 decoders. The encoder extracts representative information about various layers from shallow, fine layers to deep, coarse layers. The decoder provides semantic segmentation results on various hierarchical levels, from coarse to fine segmentation. The feature separation network has four feature selection branches, which choose the appropriate features from the extracted features and obtain the segmentation probability map of the anterior ribs, posterior rib clavicle, and all bones (ribs and clavicles). The specific operation of each module in the neural network is listed in Table 1. The number of channels of the FA layer D (number of channels of FA) selects the channel number of the corresponding encoder. FSi (*i* = 1, 2, ..., 4) represents the *i*th branch of the feature separation network.

### 2.2. Feature Reuse and Pretraining

Traditional convolutions cannot learn adequate features because of the limited dataset. However, dense connections can fully extract and use features that have been proven in classification tasks. Thus, for the encoders in our network, we no longer use traditional convolution operations but instead introduce denseBlock for adequate extraction of features and to avoid the loss of features during delivery. In addition, segmentation can be considered as a pixel-level classification. Some similar features, such as edges, may be used between the classification task and the segmentation task. Both tasks illustrate the high degree of similarity between the classification task and the segmentation task; meanwhile, the classified data are easy to tag and large in number. Thus, we add a fully connected layer after 5 encoders to obtain a classification network. The network is pretrained on the classification dataset imageNet [27]. The fully connected layer is then eliminated, and the 5 pretrained encoders are connected to the decoders to obtain the segmentation network. The segmentation network is ultimately trained on the segmentation dataset. This process prevents insufficient data from causing overfitting problems. That is, our encoder can use the structure of the pretrained DenseNet201 [28] without fully connected layers.

### 2.3. Feature Separation Network

**Figure 3:**
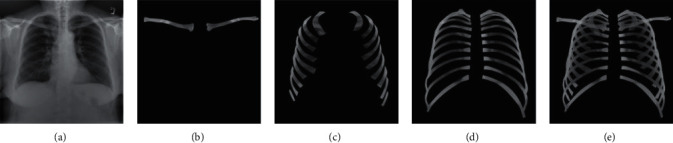
A raw chest X-ray and the corresponding parts for segmentation, including the clavicle, anterior ribs, posterior ribs, and all bones (the complete set of ribs and clavicles). (a) Chest radiograph. (b) Image of the clavicles. (c) Image of the anterior ribs. (d) Image of the posterior ribs. (e) Image of all bones.

In the chest X-ray, some pixels refer to both the anterior ribs and the posterior ribs, indicating that these pixels have multiple labels. This multilabel problem presents a challenge to classification and segmentation. Considering the practical application in medicine, we no longer split all bones on one map but convert the multilabel problem into a multitask one. That is, we mark the anterior ribs, posterior ribs, and clavicles separately and then obtain three mask images; then each pixel has only one label instead of multiple labels in one mask image. This effectively circumvents the problem of multiple tags. As shown in Figure 3, we convert a multilabel problem into a multitask problem. Consequently, our segmentation task results in four masks of the anterior ribs, posterior ribs, clavicle, and all bones (clavicles and ribs).

However, considering that a single segmentation task is converted into multiple split tasks, we need to train a model for each task. The implication is that the time cost is increased. All segmentation objects of the three images are bones with similar features. To solve this problem, we use a backbone network to extract the necessary and sufficient features first and then design a feature classification branch to distinguish the features based on the difference between them. Thus, we design the branch of the feature separation network in Figure 2 to separate the features. The total loss of the network is defined in the following equation:

(1) $$\begin{array}{c} {\text{Loss}}_{\text{total}}=\sum_{i=1}^{4}{\text{loss}}_{i}, \end{array}$$

where loss_*i*_ (*i* = 1, ..., 4) is the loss of each of the four tasks, namely, clavicle segmentation, anterior rib segmentation, posterior rib segmentation, and segmentation of all bones (clavicles and ribs). For loss_*i*_, we use cross entropy loss, as shown in the following equation:

(2) $$\begin{array}{c} {\text{Loss}}_{i}=-\frac{1}{\text{batch\_size}}\sum_{j=1}^{\text{batch\_size}}\sum_{i=1}^{n}y_{ij}\log{\hat{y}}_{ij}, \end{array}$$

where batch_size is the number of samples in a batch and *n* is the number of categories. *y*_*ij*_ is the expected output and ${\hat{y}}_{ij}$ is the actual output of the network.

The network only needs to be trained once instead of four times, which theoretically saves nearly three-fourths of the network training time. In addition, if more attention is paid to the performance of segmentation in practical applications, we can calculate the accuracy of each task after each iteration and save the best segmentation model for each task. If more attention is paid to the prediction speed, we can only save one model with the lowest total loss or the highest average accuracy.

### 2.4. Mask Coding Method

The foreground (i.e., the segmentation object) we want to segment from the X-ray chest includes the clavicle, the anterior ribs, and the posterior ribs (Figures 3(b)–3(d)). Other shadows, such as the heart, lungs, trachea, and the spine, are considered as the background. For conventional images, the network only needs to learn the characteristics of the foreground target because the background image is considerably simple. However, the background image in this task is more complex than that in a natural image. Figure 3 presents a sample of the raw image and the corresponding images that need to be segmented. Consequently, we consider enabling the network to learn not only the features of the foreground object but also those of the complex background. For each pixel, we can determine the probability, P1, that the pixel belongs to the foreground and the probability, P2, that the pixel belongs to the background. By comparison of P1 and P2, we can then determine whether the pixel is a foreground object or a background object.

**Figure 4:**
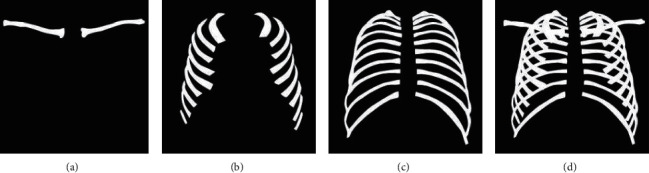
Mask images: binary maps of (a) clavicle, (b) anterior ribs, (c) posterior ribs, and (d) all bones.

In traditional encoding, if the pixel point pi is the pixel point of the segmentation object, it is encoded as 1; otherwise, the pixel is encoded as 0. However, in our mask coding mechanism, we consider one binary mask per class. That is, if this pixel is the segmentation target, it is encoded as 10; otherwise, the pixel is encoded as 01. According to this rule, we can reencode the 4 marked mask images (clavicles, anterior ribs, posterior ribs, and all bones (ribs and clavicles)). Subsequently, we can use categorical cross entropy loss, rather than binary cross entropy loss. The mask images are shown in Figure 4. We obtain the reencoded picture by negating the pixel values of the mask image and merging with the mask image.

When the network is trained, the mask images that are reencoded are regarded as the target. Therefore, in the prediction stage, the result obtained from the network is the first image representing the probability that each pixel belongs to the segmentation object. The second image is the probability that each pixel belongs to the background (other tissues and bones). On the basis of the two probability maps predicted by the network, the labels of the pixels on the final output mask picture are labels represented by a large probability value. This way also reveals that the situation where the segmentation result is not ideal due to improper selection of the threshold being avoided.

## 3. Experiments and Discussion

The data used in our research are digital chest radiograph datasets labeled by our team under the guidance of professional radiologists. The chest radiograph dataset contains 88 cases, each of which contains the corresponding mask images of clavicles, anterior ribs, and posterior ribs. The chest radiograph and corresponding mask images with a resolution of 1024 × 1024 (width × height) pixels are saved as standard PNG format. We conduct experiments by using the fourfold cross-validation method while resizing all images to 512 × 512 for the GPU limitations. Each experiment uses 22 pictures for testing and 10 pictures of the remaining 66 pictures for verification. The remaining 56 pictures are expanded to 560 pictures in every epoch by online data augmentation via operations such as rotation, shift, shear, zoom, and flip (horizontal orientation). We use the DSC, recall, precision, and the Jaccard index as indicator of segmentation.

### 3.1. Segmentation Effect of Multitask Segmentation Networks

**Figure 5:**
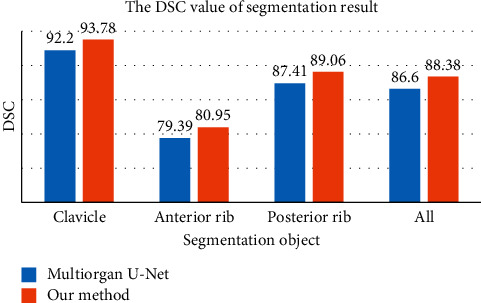
Comparison of multitask segmentation networks with respect to performance.

We compare the performance of clavicle and rib segmentation on our dataset between the proposed method and the multiorgan segmentation U-Net network [29] improved in 2018. Both techniques use the encoder-decoder structure, completing four segmentation tasks with only one training. As shown in Figure 5, the proposed method exhibits superior performance in clavicle segmentation, anterior rib segmentation, posterior rib segmentation, and segmentation of all bones.

### 3.2. Comparison of the Experimental Results of the Single-Task Segmentation Network

We used three semantic segmentation networks to perform clavicle segmentation, anterior rib segmentation, posterior rib segmentation, and segmentation of all bones (ribs + clavicle) in a chest X-ray. The semantic segmentation networks we selected are the original semantic segmentation network FCN-8s [30], the most popular segmentation network U-Net in the field of medical image segmentation [31], the latest network deeplabv3+ in natural image segmentation [32], and the proposed multitask dense connection U-Net (MDU-Net). The performance of each method is listed in Table 2. The mean and standard deviation (Std) of various metrics for each method is listed in Table 2.

As shown in Table 2, the proposed MDU-Net exhibits the best performance in segmenting all bones from a chest X-ray. Except for the precision of the proposed method in anterior rib segmentation, which is slightly lower than that of U-Net, all other indicators are higher in the proposed method than in other semantic segmentation networks. The DSC value obtained using the proposed method is 1.25, 1.36, 1.58, and 1.91 higher than that of U-Net in clavicle segmentation, anterior rib segmentation, posterior rib segmentation, and segmentation of all bones. Moreover, the DSC value of the proposed method is 3.5, 5.39, 4.4, and 5.36 higher than that of deeeplabv3+ in clavicle segmentation, anterior rib segmentation, posterior rib segmentation, and segmentation of all bones. In addition, the proposed method only needs to be trained once, whereas other networks need to be trained 4 times.

**Figure 6:**
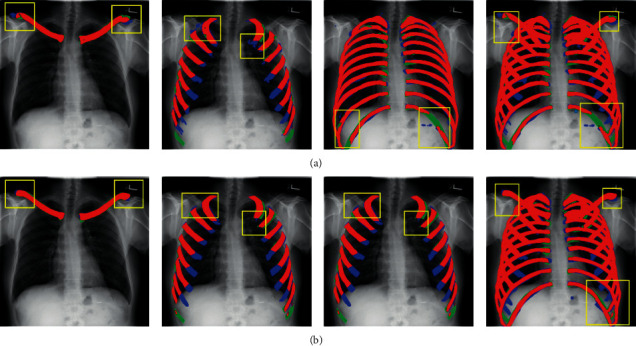
Segmentation results of U-Net and MDU-Net. The first row presents the segmentation results obtained using U-Net, and the second row shows the segmentation results obtained using MDU-Net (green, blue, and red indicate the ground-truth region, prediction region, and overlap region, respectively. Yellow boxes indicate the areas with a clear contrast).

Figure 6 compares the segmentation results of the proposed method and U-Net. The first row shows the segmentation results of U-Net, and the second row presents the segmentation results of the proposed method. Each column represents 1 segmentation task. Green, blue, and red indicate the ground-truth region, prediction region, and overlap region, respectively. We use the yellow boxes to select the areas with a clear contrast. The selected part of the box reveals that the segmentation result of the proposed method is distinctly superior to that of U-Net.

The figure shows that the ribs and clavicles segmented using the proposed method are not only relatively complete but also smoother and can be omitted after treatment. This result shows that the proposed method is more practical than U-Net and not only achieves good results but also reduces postprocessing time, which indicates that satisfactory segmentation results can be obtained within a short time.

### 3.3. Comparison of Experimental Results after Improvement

We compare the experimental results of our basic network with only the backbone network, the network with the reencoded mask as the target, the network with a multitasking mechanism, and the network after saving the multimodel. The results are listed in Table 3.

We used the results of the U-Net as a baseline. The DSC values in segmentation, obtained using the backbone network (basis) of the proposed method are 0.9, 1.09, 1.14, and 1.60 higher than the baseline in split clavicle segmentation, anterior rib segmentation, posterior rib segmentation, and segmentation of all bones, respectively. Even if the network is pretrained on datasets with different modalities and different tasks, it significantly helps improve the performance of the network. Moreover, after adding the reencoding mechanism, the feature separation network, and the method of saving a model for each task in turn, the DSC value in each segmentation task increases. After saving a model for each task, the DSC increases less because the network is not modified. Each task saves its best parameters only after the network training, and the structure of each task is exactly the same. All models are saved during the same training. Saving only one model quickens the prediction process.

## 4. Conclusion

We construct a dataset of chest X-rays and propose MDU-Net for segmenting clavicles, anterior ribs, posterior ribs, and all bones from chest radiographs. We add a dense block and use transfer learning to fully extract and use features and to prevent overfitting caused by the dataset being considerably small, increasing the generalization capability of the network. We then convert a multilabel segmentation problem into a multitasking one to reduce the difficulty of the problem and design a feature separation network to complete multiple tasks simultaneously to reduce time costs. In this process, the best model for each task can be saved to achieve the best performance for each task. Finally, we propose a mask reencoding mechanism. Using the reencoded picture as the network target can prompt the network to learn the characteristics of the background and the target simultaneously and avoid nonideal segmentation results attributable to improper selection of the threshold. The proposed method is innovative in the network structure, code method of the mask image, and time of training network. Excellent segmentation performance is achieved on our dataset. In addition, our dataset is helpful for experiments and comparison of performance in clavicle and rib segmentation.

## Figures and Tables

**Table 1:** Specific operation of each module in the neural network.

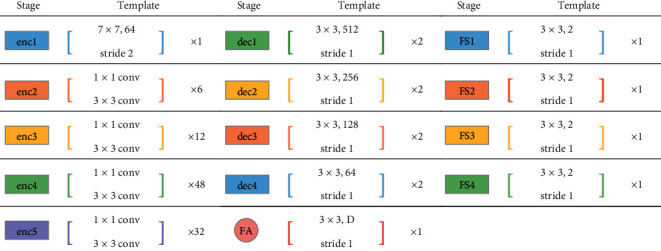

**Table 2:** Performance comparison of the single-task segmentation networks.

	Evaluation	FCN-8s [30]		Deeplabv3+ [32]		U-Net [31]		MDU_Net	
		Mean	Std	Mean	Std	Mean	Std	Mean	Std
Clavicle	DSC	90.51	3.87	90.28	4.97	92.53	5.42	**93.78**	2.21
	Precision	92.16	3.10	91.59	4.15	93.93	3.09	**94.53**	3.17
	Recall	89.26	6.14	89.47	8.05	91.59	7.45	**93.03**	3.53
	Jaccard	82.66	5.56	82.28	7.58	86.10	7.21	**88.29**	3.98

Anterior ribs	DSC	78.41	4.77	75.76	5.37	79.59	5.26	**80.95**	5.38
	Precision	83.1	10.04	77.82	10.18	**83.43**	10.4	83.18	11.02
	Recall	75.74	7.89	75.41	8.27	78.37	8.13	**81.25**	7.49
	Jaccard	64.49	6.25	60.98	6.8	66.10	6.88	**68.09**	7.2

Posterior ribs	DSC	85.39	2.67	84.66	3.44	87.48	2.83	**89.06**	2.45
	Precision	87.59	3.91	85.33	4.64	89.56	4.13	**89.69**	4.22
	Recall	83.54	4.3	84.28	4.94	85.73	4.32	**88.67**	3.62
	Jaccard	74.50	4.01	73.40	5.11	77.75	4.44	**80.28**	3.98

All	DSC	85.62	2.81	84.24	3.06	86.47	2.96	**88.38**	2.94
	Precision	88.82	4.34	86.2	5.1	89.89	4.56	**90.39**	4.71
	Recall	83.03	5.41	82.91	6.39	83.66	5.46	**86.72**	5.79
	Jaccard	74.86	4.21	72.77	5.08	76.16	4.56	**79.18**	4.68

**Table 3:** Comparison of DSC values of the improved networks.

DSC (%)	Reencoded	Multitask	Saved four models	Clavicles	Anterior ribs	Posterior ribs	All
U-Net [31]	—	—	—	92.53	79.59	87.48	86.47
MDU_Net (nonpretrained)	—	—	—	92.93	80.14	88.07	87.13
Ours (basis)	—	—	—	93.43	80.68	88.62	88.07
MDU_Net (reencoded)	√	—	—	93.64	80.75	88.74	88.16
MDU_Net (MDU-Net)	√	√	—	93.71	80.89	88.99	88.21
MDU_Net (MDU-Net^*∗*^)	√	√	√	**93.78**	**80.95**	**89.06**	**88.38**

## Data Availability

The chest radiograph data used to support the findings of this study have not been made available due to patient privacy. The chest radiograph data used to support the findings of this study are available from the corresponding author upon request.
